# The morphology of the preimaginal stages of *Cleopomiarusmicros* (Germar, 1821) (Curculionidae, Coleoptera) and notes on its biology

**DOI:** 10.3897/zookeys.798.27173

**Published:** 2018-11-21

**Authors:** Ewelina Szwaj, Jacek Łętowski, Krzysztof Pawlęga

**Affiliations:** 1 Department of Zoology, Animal Ecology and Wildlife Management, University of Life Sciences in Lublin, Akademicka 13, 20-950 Lublin, Poland University of Life Sciences in Lublin Lublin Poland

**Keywords:** Central Europe, developmental stage, host plant, mature larva, Mecinini, oligophagy, pupal stage

## Abstract

As yet little is known of the bionomics of weevils of the genus *Cleopomiarus* Pierce, 1919; current knowledge is limited to data on the morphology and biology of the preimaginal stages of certain species. This paper includes original information on the life cycle of *Cleopomiarusmicros* (Germar, 1821). It presents the morphology of the egg, last larva (L_3_) and pupa. Data on the host plant (*Jasionemontana* L.) and breeding plant (*Campanulapatula* L.) and on the oviposition and phenology of the species are updated. The anatomy of the third-stage larva of *C.micros* shares certain traits with other species of the tribe Mecinini Gistel, 1848. Comparison of the morphology of preimaginal stages of *C.micros* with those previously described for other species of the genera *Cleopomiarus* and *Miarus* Schönherr, 1826 – previously considered the same genus – reveals species differences in larval body length, colour of the body and epicranium, and chaetotaxy of head and body.

## Introduction

The tribe Mecinini Gistel, 1848 is currently represented by six genera worldwide, of which five (*Cleopomiarus* Pierce, 1919, *Gymnetron* Schoenherr, 1825, *Mecinus* Germar, 1821, *Miarus* Schoenherr, 1826 and *Rhinusa* Stephens, 1829) are known from the Palearctic fauna and one, *Rhinumiarus* Caldara, 2001, was discovered in the Neotropical realm (Caldara 2001, 2013, Caldara et al. 2014). This last genus was established based on analysis of the species *Rhinumiaruslyali* Caldara, 2001, found in Argentina, a taxon in which the external anatomy of the adult looks intermediate between species of the genera *Miarus* and *Cleopomiarus*.

The analysis of previously known adult forms and their preimaginal stages reveals a number of morphological characters common to the tribe Mecinini. In the larvae, these are as follows: head usually with 3 distinct pairs of setae (*des*) and with long, unbranched endocarina, which together with the epicranial suture extends 2/3 the length of the head; labial palpus usually with 1 segment – if there are 2 segments the basal segment is not distinctly separated; hypopharynx with usually 4, less often 2 or 6 epithelial anteromedial setae; premental rounded, less often pointed; sclerites of the prosternum joined or free, numbering 0, 2, 4 or 6; thoracic and abdominal spiracles uni- or bicameral depending on the genus, abdominal spiracles located laterally on the intersegmental membrane, tubes well defined from atrium (Emden 1938, Scherf 1964, Anderson 1973, May 1993, Marvaldi 2003, 2005, Gosik et al. 2010, Jiang and Zhang 2015). Species belonging to the Mecinini tribe are poly-, oligo- or monophages of plants of the families Campanulaceae, Scrophulariaceae and Plantaginaceae (Scherf 1964, Smreczyński 1976, May 1993, Burakowski et al. 1997, Caldara 2001, Caldara et al. 2014).

The genus *Cleopomiarus* includes 40 species dispersed throughout the world. Palearctic species (19 spp) are associated with plants of the genera *Adenophora* Fisch., *Campanula* L., *Jasione* L. and *Phyteuma* L. (Campanulaceae, Campanuloideae), whereas beetles from southern Africa and Mexico live on plants of the genera *Codonopsis* Wall., *Lightfootia* L’Hér., *Roella* L. and *Wahlenbergia* Schrad. ex Roth from the same subfamily (Campanuloideae), as well as on representatives of the genus *Lobelia* L. from the subfamily Lobelioideae (Caldara 2001, 2007, Caldara et al. 2014, Caldara and Legalov 2016). In Poland, the species thus far recorded are *Cleopomiarusdistinctus* (Boheman, 1845), *C.graminis* (Gyllenhal, 1813), *C.plantarum* (Germar, 1823) and *C.micros* (Germar, 1821). In the case of *C.plantarum*, the data come from the late 19^th^ and early 20^th^ centuries and require confirmation (Smreczyński 1976, Burakowski et al. 1997, Petryszak 2004, Wanat and Mokrzycki 2005).

As yet little is known of the bionomics of insects of the genus *Cleopomiarus*. Certain data on the morphology and biology of preimaginal stages can be found in Scherf (1964), pertaining to *C.graminis* and *C.micros*, and in Emden (1938) on *C.graminis* and *M.campanulae* (Linnaeus, 1767) and Anderson (1973) on *Cleopomiarushispidulus* (LeConte, 1876). While species identification of adult individuals poses little difficulty, systematic identification on the basis of preimaginal stages has not been possible.

In the present study the immature stages of *Cleopomiarusmicros* are described. This is a rare, psammophilous species, found only in certain areas. It inhabits sand, dunes, ruderal communities, forest clear-cuts, and thickets. It has previously been recorded in southern, western and central Europe, as far north as northern Denmark and southern Sweden. It has also been recorded in North Africa. In the literature it has been described as a monophage living exclusively in the flowers of sheep’s bit scabious, *Jasionemontana* L. (Burakowski et al. 1997, Kubisz et al. 1998, Kuśka 1999, 2001, Łętowski and Gosik 2002, Wanat 2005, Gosik 2006, Caldara et al. 2014).

The objective of the study is to describe the morphology and biology of the preimaginal stages of *Cleopomiarusmicros*, which is one of the rarest representatives of its genus in Poland.

## Material and methods

The material for the study consisted of developmental stages (egg, three larval instars and adult) of *Cleopomiarusmicros*, isolated in the laboratory from plants of the genus *Campanula* L.: *C.bononiensis* L., *C.glomerata* L., *C.patula* L., *C.persicifolia* L., *C.rapunculoides* L., *C.sibirica* L., and *C.trachelium* L., as well as from *Jasionemontana*. The plants were collected in the field in the Lublin region: Łysaków 50°45'40.96"N, 22°11'17.31"E, Podzamcze Reserve near Bychawa 51°01'24.85"N, 22°31'59.04"E, Nasutów 51°22'31.29"N, 22°31'07.32"E, Spiczyn 51°19'59.47"N, 22°44'29.63"E, Jakubowice Murowane 51°16'21.26"N, 22°38'15.52"E, Łęczna 51°18'09.7"N, 22°51'47.8"E, Ciechanki Łańcuchowskie 51°16'37.00"N, 22°55'28"E, Niedzieliska 50°41'57.07"N, 23°05'00.75"E, Kąty 50°42'21.98"N, 23°06'58.73"E, Gródek 50°46'58.18"N, 23°56'47.04"E and Czumów 50°46'28.79"N, 23°58'05.68"E. The lack of knowledge of the host plants and breeding plants of the species was the reason for the wide range of species examined from these plant genera. The selection and distribution of habitats with potential host plants were based on the results of faunistic studies in which insects of the genera *Miarus* and *Cleopomiarus* have been caught (Cmoluch 1962, 1971, Minda-Lechowska and Cmoluch 1984, Cmoluch et al. 1994, Gosik and Łętowski 2008).

Adult insects were collected by hand directly from host plants and isolated from samples collected with an entomological net in 8 × 25 series at the sampling sites over the entire growing period of the plants, from May to September, on sunny days without wind, between 10:00–16:00 local time, once a month for a period of three years (2009–2011).

Part of the individual developmental stages of these insects were fixed in 70% ethyl alcohol and a part incubated (in a ratio 1:4 – ¼ to fixation, ¾ to incubation). Breeding of preimaginal stages was carried out in Petri dishes lined with filter paper, placed in a breeding chamber with constant temperature parameters (daytime minimum 25 °C, daytime maximum 35 °C, minimum at night 15 °C, maximum at night 20 °C), humidity (60%), light intensity and duration (day 16 h, night 8 h), in order to obtain all developmental stages of the beetles (egg, three larval instars, pupa and adult) and describe their biology.

Observations of the ecology of adult beetles were also conducted in the laboratory on individuals bred together with their host plants in glass insulators at 25 °C with a 14:10 photoperiod.

Two methods were used to prepare microscope slides, as described in Łętowski (1991) and Gosik et al. (2010). Photographs of the habitat and breeding plant were taken with a NIKON Coolpix B500 camera. To prepare the drawings we used an OLYMPUS SZX12 microscope with a DP72 camera at magnifications from 200× to 400× and a TESCAN VEGA3LMU scanning microscope at magnifications from 500× to 2000×. The figures were made based on the biological preparations using COREL DRAW 18 software. The terminology of Scherf (1964), Marvaldi (1998, 1999, 2003), Skuhrovec (2004), Skuhrovec and Bogusch (2016), Wang et al. (2013) and Oberprieler et al. (2014) was used in the morphological descriptions of larva and pupa, and for chaetotaxy. The distribution and number of setae are given for half of the larval body, while the chaetotaxy of the larval head and the body of the pupa is given with respect to the whole body. Measurements of the head (following decapitation) were made on the head capsule, isolated from the body, with the mandibles closed. The dimensions of the preimaginal stages are determined from two measurements of the ova, three measurements of larval instar L_1_, 4 of L_2_, 7 of L_3_ and eight of the pupae.

## Results

### Egg

Egg white, transparent, teardrop-shaped, ca. 0.45 mm long, ca. 0.21 mm wide. Chorion surface smooth and shiny (120× magnification).

### Larva

First larval instar (L_1_) – white body, slightly transparent. Body length ca. 1 mm (0.96–1.07 mm), width ca. 0.46 mm (0.42–0.51 mm). Clearly visible pale brown head ca. 0.23 mm long (0.21–0.27 mm) and ca. 0.16 mm wide (0.13–0.20 mm). Larva with sparse setae, pedal tubercles nearly imperceptible. Anterior stemmata present.

Second larval instar (L_2_) – creamy white body, also with sparse setae. Length body ca. 2.15 mm (2.02–2.41 mm), width ca. 1.01 mm (0.90–1.09 mm). Intersegmental grooves clearly visible, pedal lobes lightly outlined. Head pale grey, length ca. 0.4 mm (0.38–0.42 mm), width ca. 0.27 mm (0.26–0.28 mm). Anterior stemmata clearly visible.

**Figure 1.**
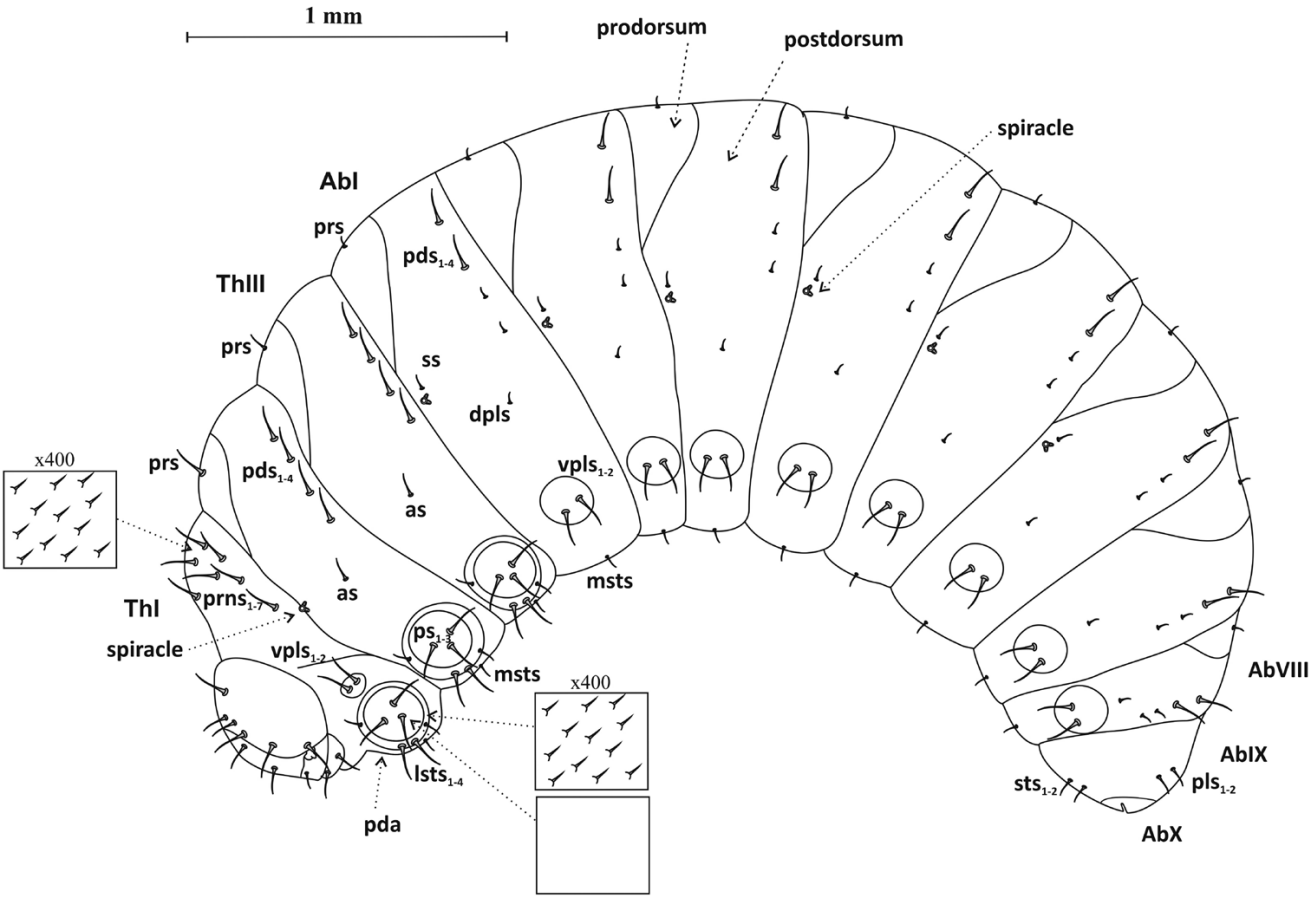
Mature larva (L_3_), lateral view: ***prns*** pronotal setae, ***vpls*** ventropleurolateral setae, ***pda*** pedal tubercle (area), ***ps*** pedal setae, ***lsts*** laterosternal setae, ***prs*** prodorsal seta, ***pds*** postdorsal setae, ***dpls*** dorsopleural setae, ***ss*** spicular setae, ***vpls*** ventropleurolateral setae, ***msts*** mesosternal seta, ***sts*** sternal setae, ***pls*** pleural setae.

Third larval instar (L_3_) – body massive, strongly curved, rounded in cross-section, creamy-white, ca. 3.41 mm long (3.12–3.73 mm) and ca. 1.51 mm wide (1.38–1.80 mm) (Figures 1, 10). Head chitinized, dark brown. Prothorax ca. 0.28 mm wide, much narrower than other two thoracic segments, ca. 0.34 mm wide. Abdominal segment I wider than others (ca. 0.49 mm), II–VII of similar width (ca. 0.38 mm). Segment VIII much narrower than others (ca. 0.23 mm), but IX markedly wider than VII – ca. 0.29 mm. Segment X reduced. Anus x-shaped. Cuticle microstructure of entire body with many small, sharply pointed cuticular structures (Figure 1). Chaetotaxy – setae of varied length, pale yellow, visible.

**Figure 2.**
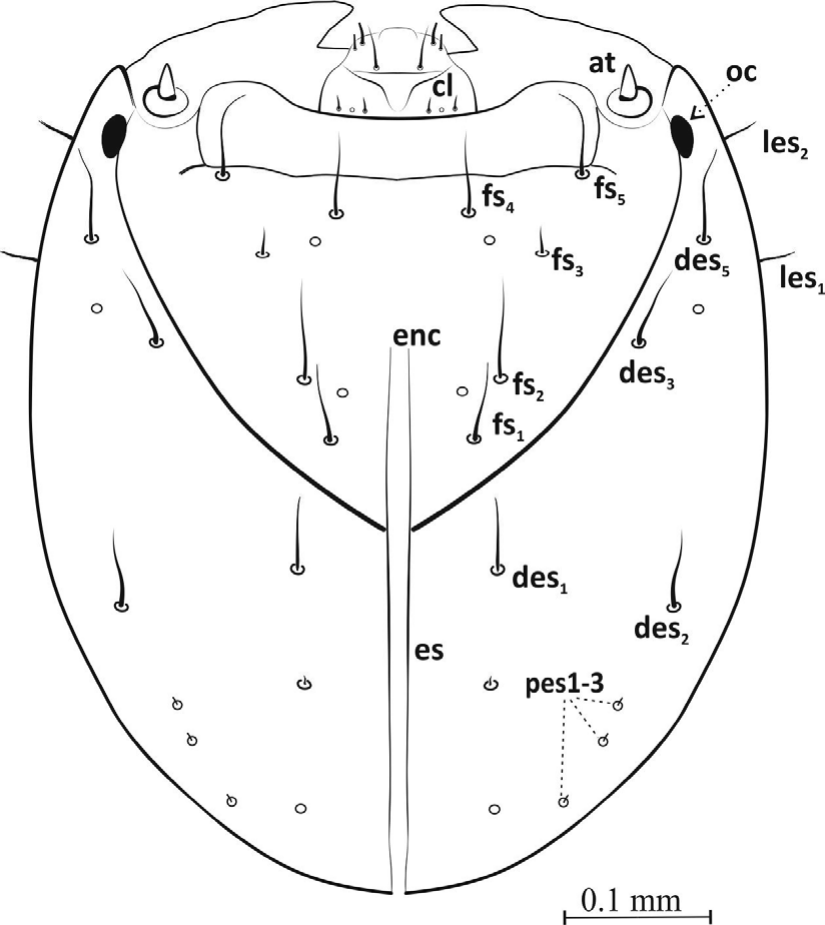
Epicranium (L_3_), dorsal view: ***les*** lateral epicranial setae, ***fs*** frontal s., ***des*** dorsal epicranial s., ***pes*** posterior epicranial s., ***cl*** clypeus, ***at*** antenna, ***oc*** ocellus, **enc** endocarina, **es** endocarina suture.

Head: Oval, dark brown, ca. 0.62 mm long (0.59–0.65 mm), ca. 0.42 mm wide (0.40–0.44 mm). Frontal suture distinct, Y-shaped, touching antennae. Endocarina distinct, long, unbranched, together with epicranial suture extends 2/3 length of head (Figure 2). On head capsule: 4 pairs of setae of varying length – the shortest *des1* and *3*, the longest *des4* and *5*, and 2 pairs of lateral setae (*les 1,2*). Seta *des2* closer to lateral edge of epicranium, *des3* and *des5* closer to frontal suture – especially *des3*. Also, 3 pairs of setae *pes1-3*. On frons 4 pairs of long hair-like setae (*fs1,2,4,5*), 1 pair of spine-like microsetae (*fs3*). Setae *fs1*,*2* near frontal suture, *fs4,5* near epistomal suture and two pairs of sensillae (Figure 2).

**Figure 3.**
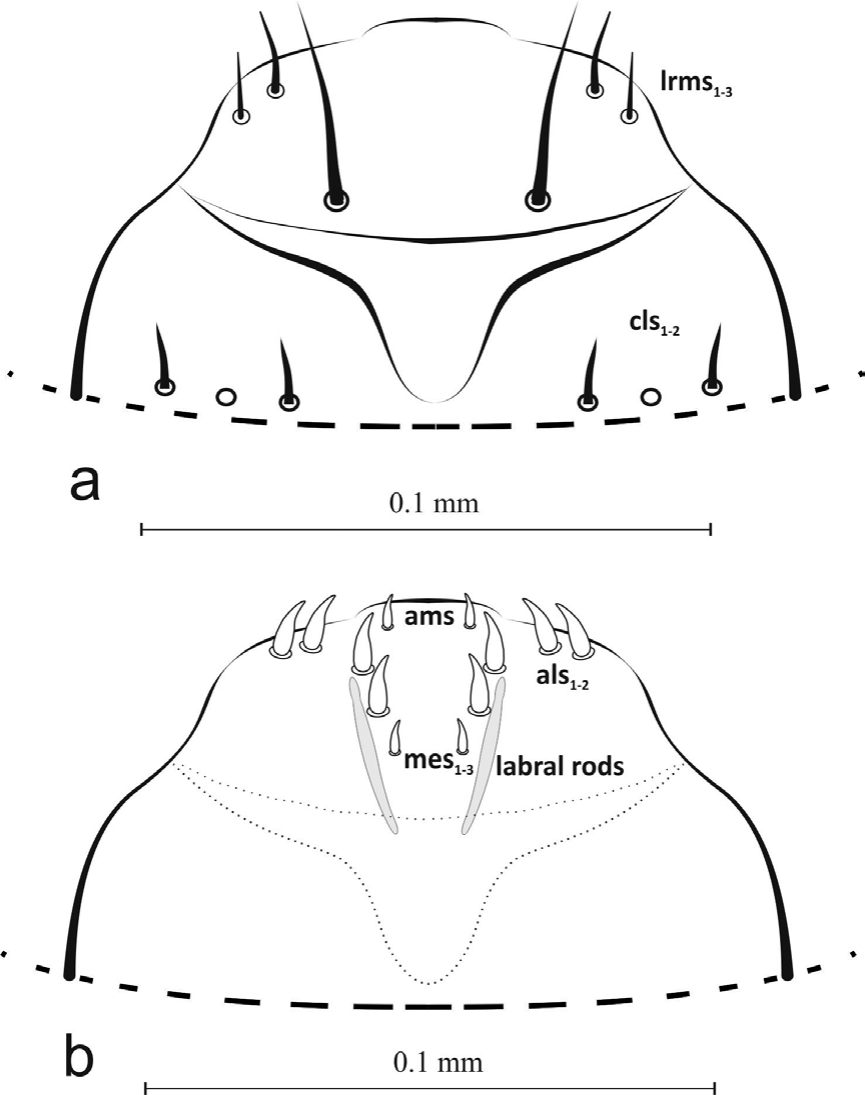
Labrum and clypeus (L_3_): dorsal view (**a**) ***lrms*** labral setae, ***cls*** clypeus s., ventral view (**b**) ***mes*** median s., ***ams*** anteromedial s., ***als*** anterolateral s..

Clypeus wider than long (ca. 0.11 mm × 0.03 mm), trapezium-shaped. Anterior margin concave, 2 pairs of short, sharp, thorn-shaped microsetae (*cls1*,*2*) along posterior margin, between them sensilla (Figures 3a, 14). Antennae (*at*) at end of frontal suture, clearly visible, sensorium slightly elongated, finger-shaped, with 4 sensilla at base (Figure 16). Anterior stemmata visible. Tentorial bridge present.

**Figure 4.**
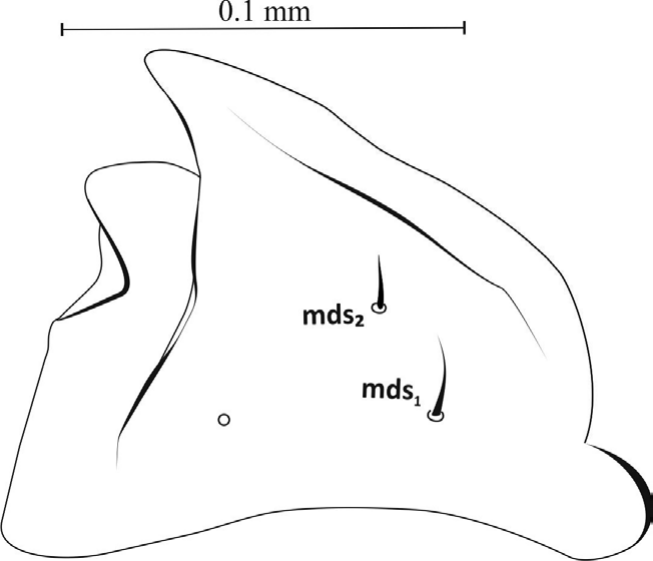
Mandible (L_3_), right: ***mds*** – dorsal malae setae.

**Figure 5.**
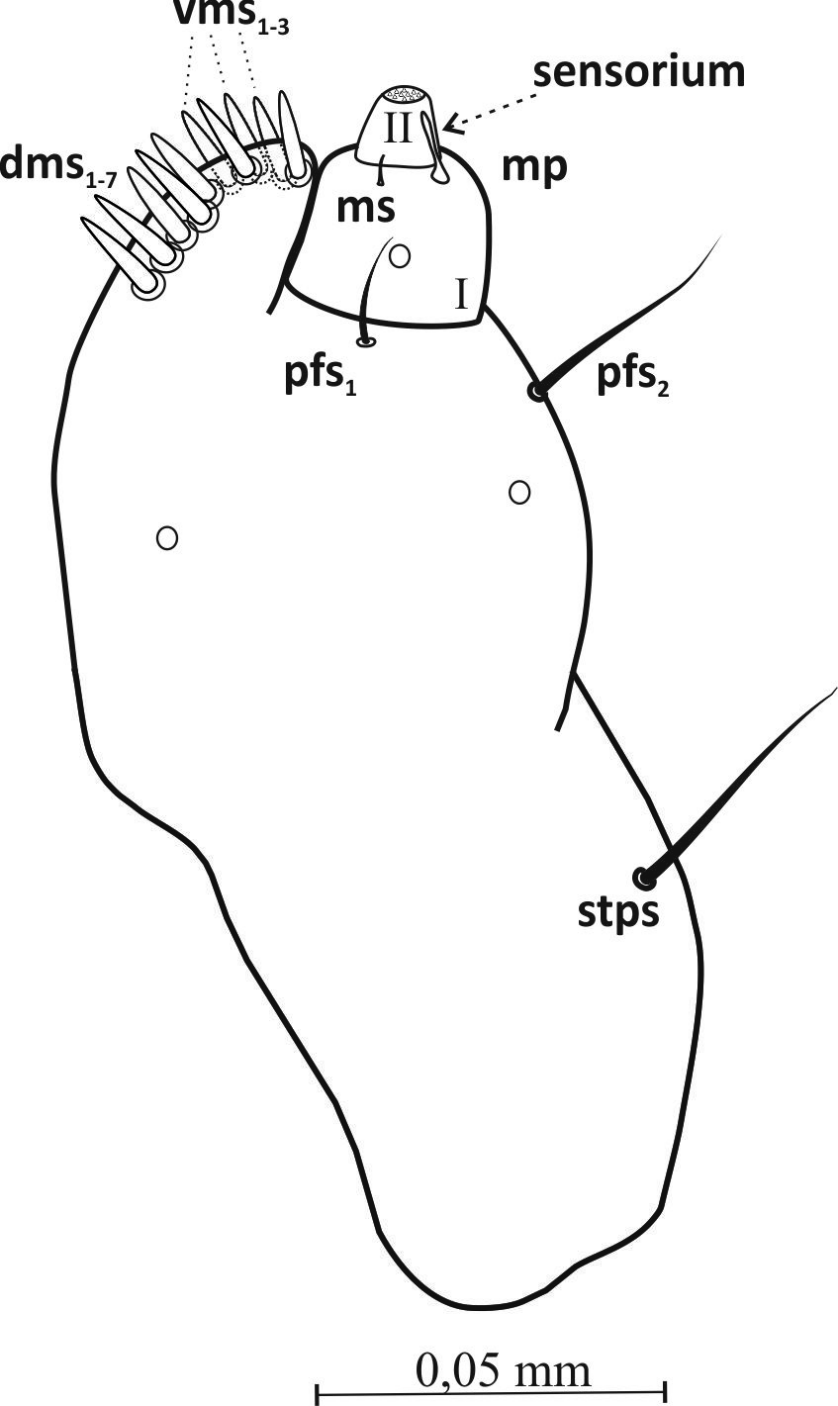
Maxillae (L_3_), dorsal (a) and ventral (b) view: ***dms*** dorsal maxillary setae, **vms** ventral maxillary s., ***pfs*** palpiferal s., ***sts*** stipal s., ***mps*** maxillary palpus s., ***mp*** maxillary palpus, ***ms*** microseta.

Mouthparts: Labrum more then 2× wider than long (ca. 0.06 mm × 0.02 mm), with 3 pairs of setae (*lrms1*-*3*) of varying length – *lrms3* the shortest and *lrm1* the longest (Figures 3a, 14). Epipharynx with 1 pair of finger-shaped setae (*als1*) and 3 pairs of *mes*, 2 pairs of *ams* and visible long labral rods (Figure 3b). Mandible large, highly sclerotized, dark brown, apically bidentate (Figure 4); teeth of equal size, with apices usually worn down in mature larvae; inner margin with triangular tooth at half-height from base. Mandibular setae *mds1*,*2* of similar length. Maxilla – stipes (*st*) quite wide, with 3 distinct, hair-like setae (Figure 5). Setae *pfs1* and *stps* long, of approx. equal length, *pfs2* half their length and located at base of palpus. Malar part of maxilla with clearly visible, finger-shaped setae of equal length: 7 on dorsal side (*dms1-7*) and 3 on ventral side (*vms1-3*) (Figure 15). Maxillary palpus (*mp*) with 2 segments, distal segment markedly smaller than basal segment, with 10 nodular cuticular tubercles situated apically (Figure 15). On basal segment sensorium and 1 microseta (*ms*) (Figure 5).

**Figure. 6.**
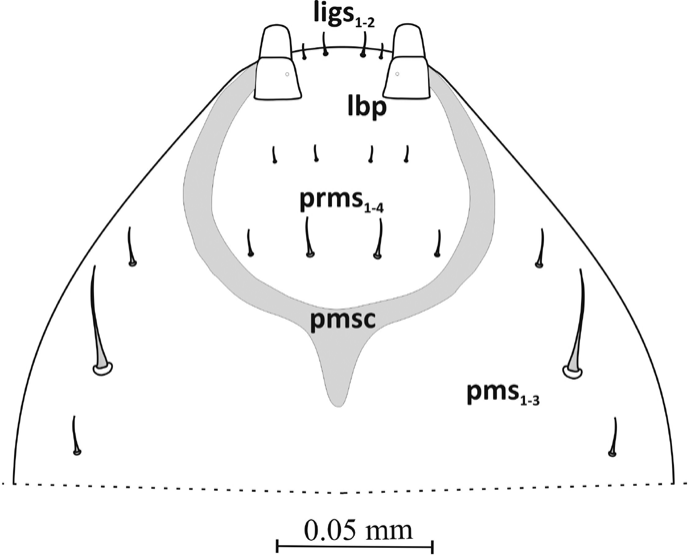
Labium (L_3_): ***prms*** prelabium setae, ***pms*** postlabium s., ***pmsc*** premental sclerite, ***ligs*** ligular s., ***lbp*** labial palpus.

Labium – prementum (prms) nearly oval, with 2 pairs thorn-shaped microsetae (*ligs1*,*2*) near anterior margin and 4 pairs of hair-like micro- and macrosetae arranged in 2 rows in middle and at base (*prlbs1-4*). Prementum base (*prmsc*) rounded, weakly sclerotized. Labial palpus (*lba*) longer than wide with 2 segments of similar length, basal segment weakly separated from distal (Figure 17). Postmentum (*pms*) with 3 pairs of setae along the sides – middle setae (*pslbs2*) at least 3 times longer than others (Figure 6).

Thorax: Prothorax with 7 long setae (*prns*) on dorsal side, 2 long ventropleurolateral setae (*vpls*), 7 setae on pedal tubercle (*pda*), including 2 micro- and 5 macrosetae – *ps1-3*, *lsts1-4*, and 1 ventral seta (*msts*) (Figure 1). Mesothorax with 1 long prodorsal seta (*prs*), 4 long postdorsal setae (*pds1-4*), 1 microseta *dpls*, 7 setae on pedal tubercle (*pda*), including 2 micro- and 5 macrosetae, and 1 ventral seta (*msts*). Distribution and shape of metathoracic setae as on mesothorax, except for much shorter dorsal seta (*prs*). On all thoracic segments pedal tubercles visible, with distinctive cuticle structure. Centre of tubercles with smooth cuticle and 3 setae, edge zone of tubercles with thorn-like structure and remaining setae (*lsts1*-*4*) (Figure 1). All thoracic macrosetae about 2 times longer than microsetae. Thoracic spiracle bicameral, 6-ringed, located intersegmentally, between ThI and ThII.

Abdomen: Abdominal segments I–VII with 1 prodorsal microseta (*prs*), 4 (2 macro- and 2 micro-) postdorsal setae (*pds*), 1 spicular seta (*ss*), 1 dorsopleurolateral microseta (*dpls*), 2 ventropleurolateral setae (*vpl*s) and 1 mesosternal seta (*msts*). Macrosetae clearly extend beyond outline of body and are several times longer than microsetae. Segment VIII with similar chaetotaxy as previous segment. Abdominal segment IX with 2 dorsal microsetae (*pls*) and 2 ventral microsetae (*sts*) of similar length (Figure 1). All spiracles (6) bicameral, 6-ringed, positioned size.

### Pupa

The female and male morphology is externally very similar at the pupal stage, with a significant difference only in the length of the rostrum, which is longer in the female and its apex extends to the middle of the second tarsal segment of the prolegs; in the male the rostrum is short and its apex extends to the end of the first segment.

**Figure 7.**
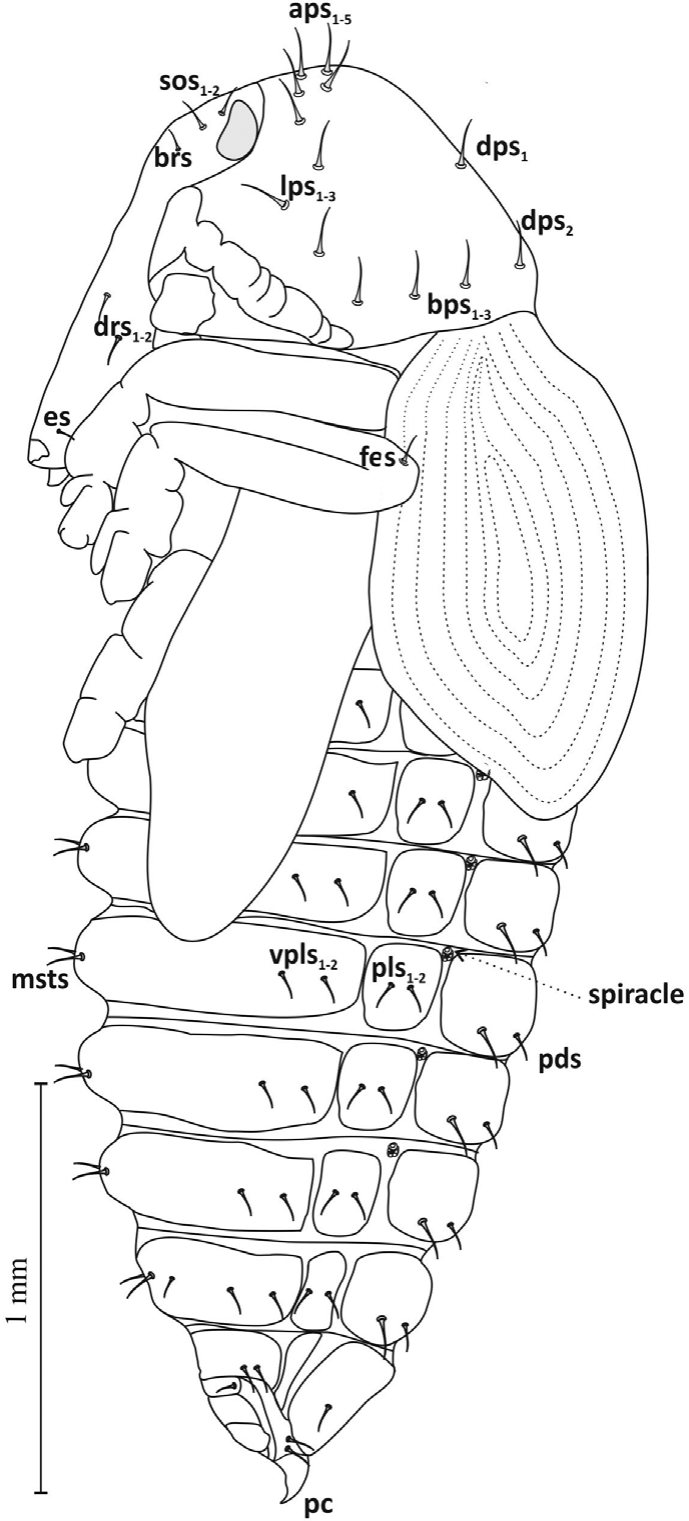
Pupa, lateral view: ***aps*** apical pronotal setae, ***lps*** lateral pronotal s., ***dps*** discal pronotal s., **bps** basal pronotal s., ***sos*** supraorbital s., ***brs*** basirostral s., ***drs*** distriostral s., **es** epistomal s., ***fes*** femoral s., **msts** mediosternal s., **pls** pleural s., **vpls** ventropleural s., **pds** postdorsal s., ***pc*** urogomphi (pseudocerci).

**Figure 8.**
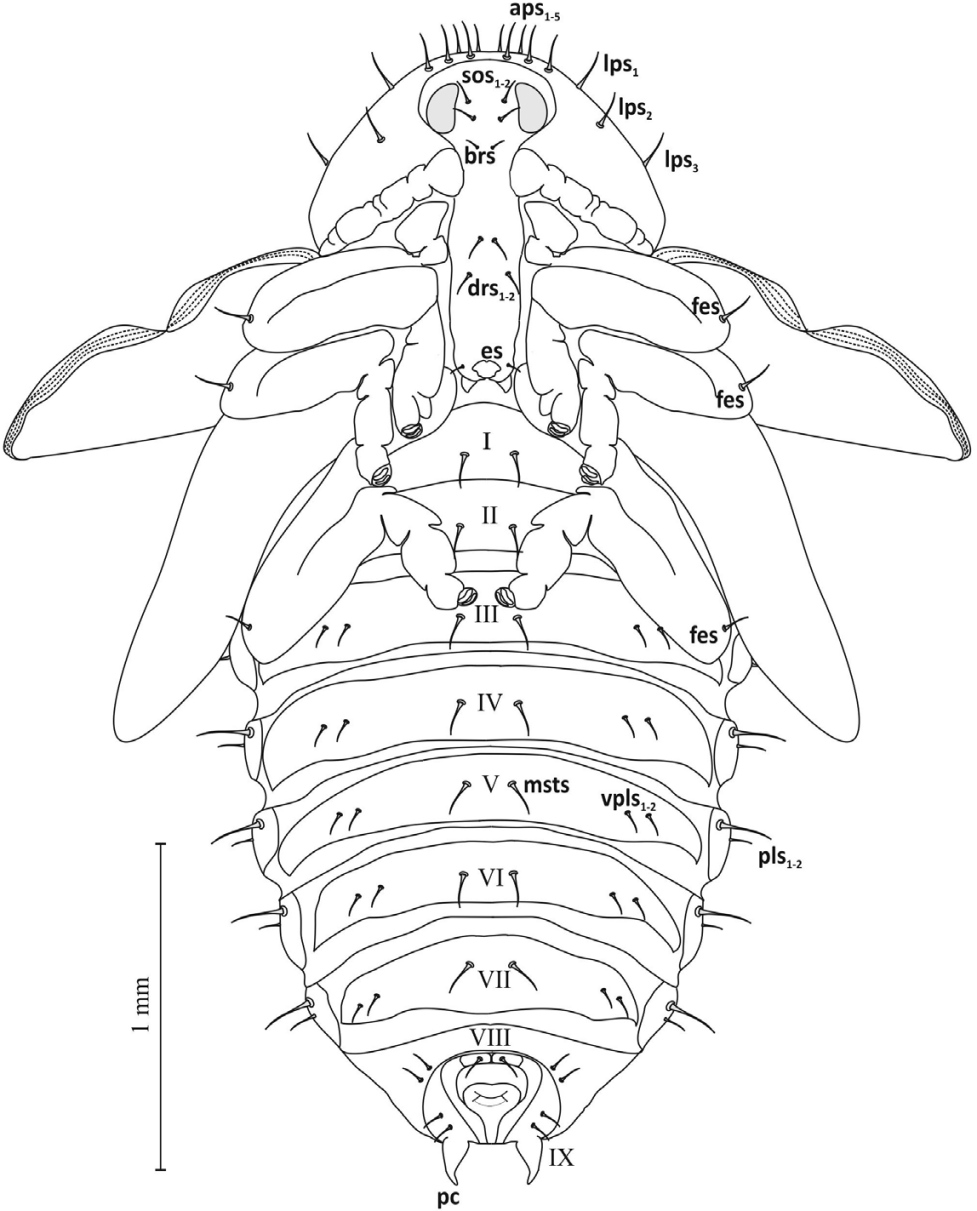
Pupa, ventral view.

**Figure 9.**
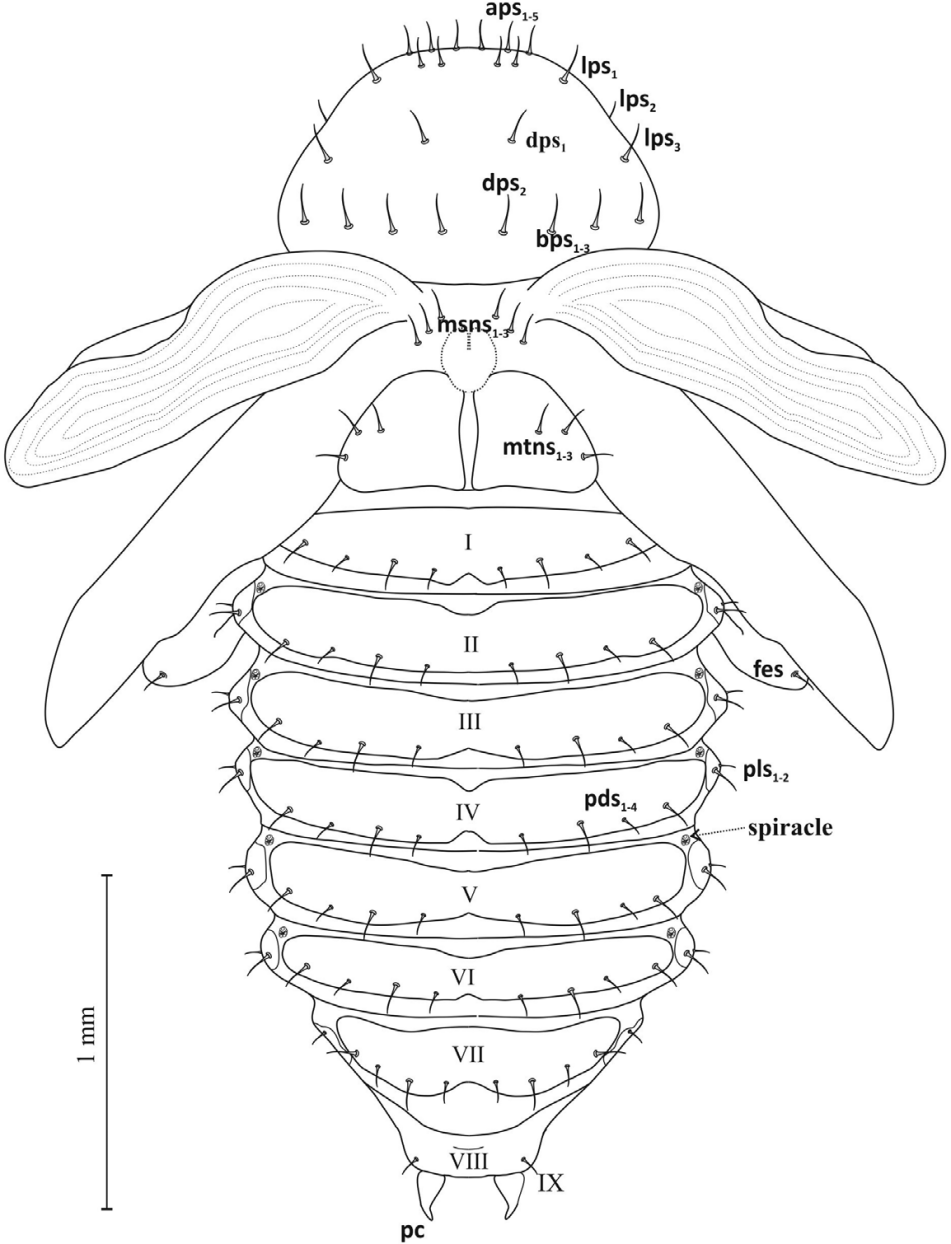
Pupa, dorsal view: **msns** mesonotal setae, **mtns** metanotal s.

Body: Length ca. 3.33 mm (2.95–3.53 mm), width ca. 1.41 mm (1.15–1.67), colour dark brown (Figures 7–9, 11).

Head: Head capsule with 2 pairs of *sos.* Female rostrum longer (ca. 0.68 mm), broader in middle with 1 pair *brs*, 2 pairs *drs* and 1 pair *es*, and extends to end of segment II of first pair of legs. All setae on rostrum short, spinescent (Figures 7, 8).

Thorax: Pronotum broad (width ca. 1.12 mm, length ca. 0.65 mm) with 5 pairs of apical pronotal setae (*aps1*-*5*), 3 pairs of lateral pronotal setae (*lps1*-*3*), 2 pairs of discal pronotal setae *dps* and 3 pairs of basal pronotal setae (*bps1-3*) (Figures 7–9). Mesonotum shorter than metanotum (length ca. 0.26 and ca. 0.36 mm) (Figure 9). On dorsal part of meso- and metanotum 3 pairs each of spinescent setae, *msns* and *mtns*. All setae of pro-, meso- and metanotum of equal length.

Abdomen: Tergites of abdominal segments I–VII of similar width, gradually narrowing slightly towards rear, with 4 pairs of setae *pds* (2 macro- and 2 micro-) arranged alternately, parallel to posterior margin of segment (Figure 9). On tergite VIII 1 pair of short microsetae. Macrosetae of tergites longer than microsetae by more than half. Sternites I–VII with 1 pair of larger *msts* and 2 pairs of smaller *vpls*, on sternite VIII 2 pairs of microsetae (Figures 7, 8). Macro- and microsetae of sternites somewhat longer than macro- and microsetae of tergites, but in similar proportions. Segment IX with short, pointed, slightly curved urogomphi (*pseudocerci*), and 3 pairs of setae (Figure 8). Pleurites of segments I–VII with 2 setae *dpls* (Figure 7). Spiracles (*spiracle*) located between tergite and pleurite, in upper part of border, clearly visible on segments I–VI, on others absent (Figures 7, 9).

### Biological information

Analysis of eight plant species of the family Campanulaceae revealed that *Cleopomiarusmicros* uses *Jasionemontana* as a food source for the imago and *Campanulapatula* for breeding larvae. The presence of these two plants at the same habitat provides ideal conditions for the occurrence of the species (Figure 12). Only at the study site in Nasutów were these plants present at the same time. Adults of *Cleopomiarusmicros* hibernate in the soil and come out in late spring. The first adults were caught in the second half of May. A total of 35 specimens were caught, but even this small number allowed for important conclusions to be formulated:

– Adult insects feed on sheep’s bit scabious (*Jasionemontana*).

– Following copulation at the beginning of June, females choose a different plant species of the same family – spreading bellflower (*Campanulapatula*) – to lay their eggs.

– Single eggs are laid on the wall of the ovary (seed chamber). Unlike in other beetle species of this genus, delicate traces of oviposition appear on the plant’s epiderm. To lay an egg the female uses a natural hollow on the exterior of the seed chamber, which she gently gnaws and enlarges (Figure 13).

– The L_1_ larva bores a tunnel to the inside of the chamber. Subsequent larval instars (L_2_ and L_3_) eat the contents of the seed chamber. After seven days of growth the L_3_ larva transforms into a pale brown pupa, and after another four days into an imago. As observed, the adult leaves the seed through cracks formed during its drying prior to germination.

– One generation per year was noted in the species.

– The insect does not cause cecidia in the breeding plant.

**Figures 10–17.**
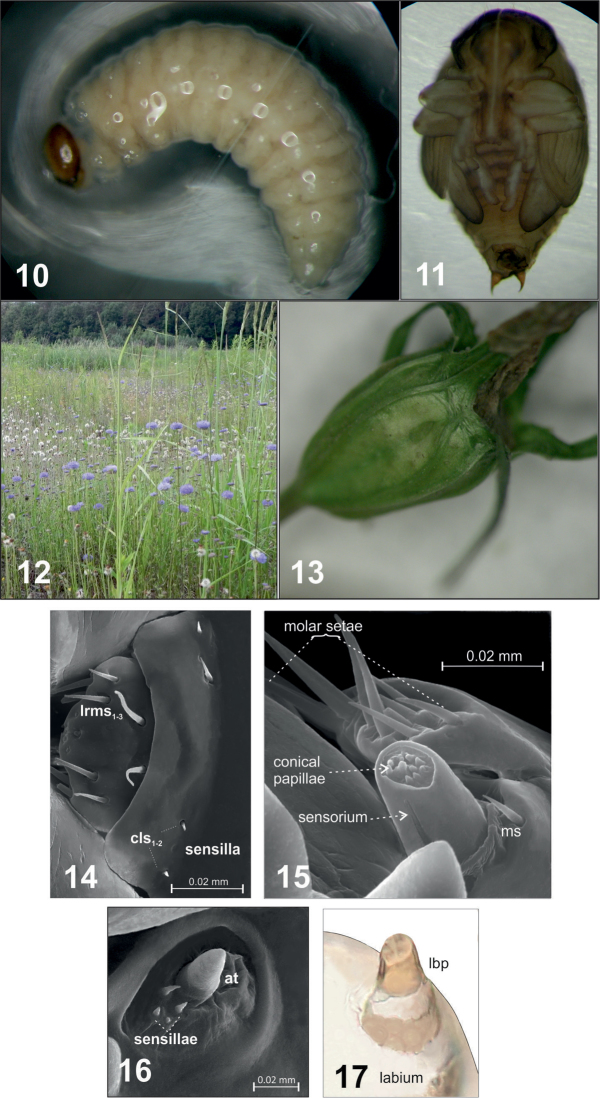
*Cleopomiarusmicros* (Germar, 1821): **10** mature larva **11** pupa **12** the environment of the species **13** place lay eggs on the plant breeding **14** labrum and clypeus (L_3_) (dorsal view): ***lrms*** labral setae, ***cls*** clypeus s. **15** apical part of the maxilla: ***ms*** microseta **16** antennae (***at***) **17** labial palpus (***lbp***).

## Discussion and conclusions

The results of the study, the first morphological description of the preimaginal stages of the species *Cleopomiarusmicros*, serve to verify and supplement previous scant data on the biology of the species. Previous data on the morphology of the preimaginal stages of representatives of the genus *Cleopomiarus* concern only *C.graminis*, *C.hispidulus*, and *Miaruscampanulae*, which in the old taxonomic system belonged to the genus *Miarus* (Emden 1938, Scherf 1964, Anderson 1973, Caldara and Legalov 2016).

The morphology of the L_3_ larvae and pupa of *C.micros* does not differ from the typical characters of preimaginal stages of weevils of the tribe Mecinini (Emden 1938, Scherf 1964, Anderson 1973, Marvaldi 2003, 2005, Marvaldi and Lanteri 2005, Gosik 2010, Jiang and Zhang 2015). The morphology of mature *C.micros* larva is similar to the description of the characters of the third-stage larvae of the tribe presented by Emden (1938), including pronounced convexity of the body, a long, unbranched endocarina, which together with the epicranial suture extends two-thirds the length of the head, and an intersegmental thoracic spiracle.

Certain anatomical traits of *C.micros* are common to species of Mecinini tribe, but less frequent. As mentioned in the Introduction, the head of species of this tribe usually has 3 pairs of *des.* In *C.micros* there are 4 pairs, as in *Rhinusabipustulata* (Rossi, 1792) or *Gymnetronmiyoshii* Miyoshi, 1922 (Gosik 2010, Jiang and Zhang 2015). In Mecinini, the labial palpus usually has a single segment; if there are two segments the basal segment is not distinctly separated. This is the case with *C.micros*, which has a two-segmented palpus. The presence of a two-segmented labial palpus seems to be a character common to species of the genus *Cleopomiarus*. This is confirmed by species such as *Cleopomiarusdistinctus* and *C.graminis* (the authors’ unpublished data). A two-segmented labial palpus is also present in species of the genus *Miarus* (*M.campanulae* and *M.ajugae*) (Scherf 1964, the authors’ unpublished data). Another character present in *C.micros*, which is common to species of the Mecinini tribe but occurs less frequently, is the presence of two anteromedial setae (*ams*) on the epipharynx. The same number of *ams* is found in *Gymnetronantirrhini*, *G.lineariae* and *G.villosulum* (Emden 1938, Scherf 1964). Analysis of the anatomical characters of *C.micros* in comparison with other species of the Mecinini tribe suggests that it is most similar to species of the genus *Miarus* (Emden 1938, Scherf 1964, Anderson 1973).

Comparison of the morphology of *C.micros* with previously described preimaginal stages (L_3_ and pupa) of species of the genera *Cleopomiarus* and *Miarus* (previously the same genus) on the basis of previously described features reveals species differences in larval body length, the colour of the body and epicranium, and the chaetotaxy of the head and body (Emden 1938, Scherf 1964). Among the species compared, the larva of *C.micros* has the shortest body (on average 3.41 mm), and *C.graminis* has the longest (up to 5 mm). In *M.campanulae*, as distinct from *M.ajugae* (Herbst, 1795), the average larval length is 4.75 mm (our own data). The colour of the larvae ranges from white in *M.campanulae* to cream-coloured in *C.micros* to yellowish-white in *C.graminis*. The head in individual species is of various shades of brown: yellowish-brown in *C.graminis*, dark brown in *C.micros* and black-brown in *M.campanulae* (Scherf 1964). The differences in the chaetotaxy of the body and the form of the mouthparts are presented in Table 1. The present research made it possible to discover, supplement and describe the chaetotaxy of many parts of the body of *C.micros*.

**Table 1.** Diagnostic features of the mature larvae of *Cleopomiarusmicros*, *C.graminis*, *C.hispidulus* and *Miaruscampanulae* (‘-’ indicates lack of descriptive data).

Traits	Character	* Cleopomiarus micros *	*Cleopomiarusgraminis* (Emden 1938, Scherf 1964)	*Cleopomiarushispidulus* (Anderson 1973)	*Miaruscampanulae* (Emden 1938, Scherf 1964)
Head	Epicranium	*pes1-3*; *des1*-*3,5; des4* absent; *les1*-*2*; *fs1-6*; *oc* present; *cls1*-*2* and 1 *sa*	–	–	–
	Antennal sensorium	slightly elongated, finger-shaped, with 4 *sa*	short, conical	elongated	finger-shaped
Mouthparts	Mandible	2 apical teeth, incisive margin with tooth, *mds1-2*, 1 *sa*	2 apical teeth, incisive molar edge with tooth	–	2 apical teeth, *mds1-2*
	Labrum	*lrms1-3*	*lrms1-3*	–	*lrms1-3*
	Epipharynx	1 pair *ams*, *als1*, 3 pair *mes*, long epipharyngeal rods present	*ams1-3*, *als1-3*, 2 pair *eps* (*mes*) short epipharyngeal rods present	–	–
	Maxilla	1 *stps*, *pfs1*-*2*, 2 *sa*, *mxp2* segments, basal with 1 *sa* and accessory process, apical segment with 1 *sa*; *dms1-7* and *vms1-3*	*mxp2* segments, 8 maxilla setae (*dms* and *vms*)	–	*mxp2* segments, maxilla setae present
	Labium	*pms1-3*; premental sclerite „Y” shaped; *prms1-4*; *ligs1-2*; *lbp2* segments – *lbp* longer then wide, basal segment with 1 sa, segments of similar length, basal segment fairly well defined	premental sclerite rounded, *lbp* 2 segments – *lbp* longer then wide, 2^nd^ segments as long as wide, basal segment rather well defined	–	premental sclerite rounded, *lbp2* segments – *lbp* as long as wide, 2^nd^ segments distinctly longer then wide, basal segment not well defined
Thorax	Th1	*prns1-7*, *vpls1-2*, *ps1-3, lsts1-4*, 1 *msts*	*pda* with 3 setae (*ps1-3*)	–	a few setae on dorsal plate, *pda* with 3 setae (*ps1-3*), *vpls* with 1 setae, 1 *msts*
	Th2	1 *prs, pds1-4*, 1 *as, ps1-3, lsts1-4*, 1*msts*, 1 *as*	*pda* with 3 setae (*ps1-3*)	–	1 pair *prs*, *pds1-4, ps1-3*, *as1-2*
	Th3	same as Th2	*pda* with 3 setae (*ps1-3*)	–	same as Th2
Abdomen	Abd I–VII	1 *prs, pds1-4*, 1 *dpls, vpls1-2*, 1 *msts*	–	–	–
	Abd VIII	same as AbI–VII	–	–	–
	Abd IX	*ds1-2, sts1-2*	–	–	–
	Abd X	anal x-shaped, without setae	anal x-shaped	anal x-shaped	anal x-shaped
Spiracles	Thorax	bicameral	bicameral	bicameral	bicameral
	Abdomen	bicameral	bicameral	bicameral	bicameral

Differences between the species analysed were also noted in the size and colour of the pupal body. In *C.micros* the body is 2.95–3.53 mm long and dark brown, whereas the yellow-white pupa of *C.graminis* reaches a length of 3.4–5.0 mm and the body of the *M.campanulae* pupa is 2.5–3.0 mm long and white (Scherf 1964, Anderson 1973). The differences in the chaetotaxy of the body and the shape of the pseudocerci are presented in Table 2.

The development of the Mecinini species is correlated with the phenology of the breeding and host plants and is strongly dependent on the environmental conditions prevailing at a given site. In the case of *C.micros*, a necessary condition for the presence of the beetle is the co-occurrence of plants of the species *Campanulapatula*, in which it develops, and *Jasionemontana*, which constitutes the food base for adults. This was confirmed by the observations in the field and experiments in the laboratory. The species was not found when only one of the listed plant species was present in the environment. Among the species from genus of *Miarus* and *Cleopomiarus* so far studied, only *C.micros* develops in such a way – development in a one species of plant and feeding on another. This indicates that this species is oligophagous. Until now, it would have been described as a monophag (Scherf 1964). Probably, the development in *Campanulamontana* is conditioned by the appropriate size of the ovary and the abundance of seeds.

Understanding of the morphology of the adult larva of the described species allows for the recognition of potential generic features for the labial palp of *Cleopomiarus* species. They are: the ratio of length to its width – the labial palp is longer than wider, the apex is clearly longer than wider, and the basic segment is pronounced. Morphological descriptions of the next species of the genera *Miarus* and *Cleopomiarus* will probably allow other generic features to be distinguished, and, in turn, for the production of a key to identify mature larvae.

The data presented herein provide new information on the biology and ecology of the species. Previous data covered only its living environment, host plant and the number of generations per year (Scherf 1964, Burakowski et al. 1997).

**Table 2.** Diagnostic features of pupae of *Cleopomiarusmicros*, *C.graminis* and *Miaruscampanulae* (‘-’ indicates lack of descriptive data).

Traits		* Cleopomiarus micros *	*C.graminis* (Scherf 1964)	*Miaruscampanulae* (Scherf 1964)
Head		*sos1-2, brs1, drs1-2, es1*	–	*os1-2, sos1, vs1, rs1* (*drs*)
Thorax (one side)	Pronotum	*aps1-5, lps1-3, dps1-2, bps1-3*	–	as1-3(*aps*), *ls1-2* (*lps*), *ds1* (*dps*), *pls1* (*bps*)
	Mesonotum	*msns1-3*	–	3 *sas* (*msns*)
	Metanotum	*mtns1-3*	–	3 *sas* (*mtns*)
Abdomen (one side)		*pds1-4, dpls1-2*,	–	4 pairs of setae
Legs		1 *fes*	–	1 *fes*
Pseudocerci		pointed, slightly curved	pointed, curved	pointed, massive, arched
